# Selected Dietary Nutrients and the Prevalence of Metabolic Syndrome in Adult Males and Females in Saudi Arabia: A Pilot Study

**DOI:** 10.3390/nu5114587

**Published:** 2013-11-18

**Authors:** Nasser M. Al-Daghri, Nasiruddin Khan, Khalid M. Alkharfy, Omar S. Al-Attas, Majed S. Alokail, Hanan A. Alfawaz, Abdulaziz Alothman, Paul M. Vanhoutte

**Affiliations:** 1 Center of Excellence in Biotechnology Research, King Saud University, Riyadh 11451, Saudi Arabia; E-Mails: alkharfy@ksu.edu.sa (K.M.A.); omrattas@ksu.edu.sa (O.S.A.-A.); msa85@yahoo.co.uk (M.S.A.); 2 Biomarkers Research Program, Biochemistry Department, College of Science, King Saud University, Riyadh 11451, Saudi Arabia; E-Mail: nasiruddin2006@gmail.com; 3 Clinical Pharmacy Department, College of Pharmacy, King Saud University, Riyadh 11451, Saudi Arabia; 4 Prince Mutaib Chair for Biomarkers of Osteoporosis, Biochemistry Department, King Saud University, Riyadh 11451, Saudi Arabia; E-Mails: halfawaz@ksu.edu.sa (H.A.A.); amothman@ksu.edu.sa (A.A.); 5 Department of Food Science & Nutrition, College of Food Science & Agriculture, King Saud University, Riyadh 11451, Saudi Arabia; 6 College of Applied Medical Sciences, King Saud University, Riyadh 11451, Saudi Arabia; 7 Department of Pharmacology and Pharmacy, Li KaShing Faculty of Medicine, 21 Sassoon Road, Hong Kong, China; E-Mail: vanhoutt@hku.hk

**Keywords:** dietary micronutrients, metabolic syndrome, adult females, vitamin, Saudi Arabia

## Abstract

During the last decade, the rapid economic development in Saudi Arabia resulted in an unbalanced dietary intake pattern within the general population. Consequently, metabolic syndrome was also documented to be highly prevalent in the Middle-East region. We aimed to examine the relationship between selected dietary nutrient intakes and the prevalence of metabolic syndrome in the general adult population of Riyadh, Saudi Arabia. In this cross-sectional study, 185 adult Saudis aged 19 to 60 years (87 males and 98 females (mean age 35.6 ± 13.2 and 37.6 ± 11.7 years, respectively)) were included. The criteria for metabolic syndrome were based on the International Diabetes Foundation (IDF) criteria, and the dietary food intake was assessed by two 24-h dietary recall methods. The odd ratios (ORs) of metabolic syndrome risk across quartiles of selected dietary nutrients were significantly lower for carbohydrates and proteins, as well as for vitamins A, C, E and K, calcium, zinc and magnesium (*p* < 0.05 for all) in the female group with metabolic syndrome than those without. The pattern of daily dietary intake of selected nutrients among the general population of Saudi Arabia raises concern, and this dietary imbalance could increase the risk of metabolic syndrome, particularly in adult Saudi females.

## 1. Introduction

Metabolic syndrome represents a combination of several risk factors encompassing central obesity, insulin resistance, dyslipidemia and hypertension [1,2]. These risk factors also contribute largely towards the development of type 2 diabetes mellitus and cardiovascular diseases (CVDs) [3,4]. The National Cholesterol Education Program Adult Treatment Panel III (NCEP ATP III) documented that about 50 to 75 million people in the USA harbor metabolic syndrome [5], while in Europe, the prevalence in men and women is 15.7% and 14.2%, respectively [6].

Dietary factors have a major influence on metabolic syndrome. Indeed, nutritional imbalance due to high energy, fat and cholesterol intakes are considered to be a risk factor for the occurrence of this syndrome [7]. In addition to the findings of NCEP ATP III, several studies also suggest that lifestyle modifications could be an important element for reducing and managing metabolic syndrome risk factors [8,9]. Several clinical and epidemiological studies suggest that among the therapeutic lifestyle changes, dietary factors could play a very important beneficial role in combating several chronic diseases [10,11]. It has also been shown that the various risk factors contributing to metabolic syndrome differ between genders and in different countries [12].

In recent decades, Saudi Arabia has rapidly developed economically and socially. This pace of change has affected the general dietary intake pattern of the population, with a preference towards increased consumption of energy dense and processed foods that are high in fat, sugar and salt. This fast-paced lifestyle seems to be a major factor in the growing epidemic of non-communicable diseases in the Arab region [13,14]. Unhealthy diets are considered major causes of diseases, such as cardiovascular disease and type 2 diabetes mellitus, contributing substantially to the global burden of diseases and mortality in Middle-East countries [15,16]. In Saudi Arabia, 66% of adult men and 71% of adult women are either overweight or obese [17]. These countries spend nearly 5.6 billion USD on diabetes-related healthcare [18].

The available data suggest that metabolic syndrome is an increasingly common problem in the Arab population, and the estimates of its prevalence vary from 20.8% to 40% [19,20,21]. In 2005, Al-Nozha and colleagues [19] reported that the prevalence of metabolic syndrome was almost 40% in Saudi Arabia. In addition, several epidemiological studies also demonstrated an increase in the prevalence of metabolic syndrome in other Middle-Eastern countries [22,23,24]. A recent study by Al-Daghri and colleagues [25] in Saudi adults observed that the prevalence of full metabolic syndrome remains high, but has considerably decreased, reaching 37% as compared to the previously recorded 44.1% [19]. However, among the metabolic syndrome components, low high-density lipoprotein (HDL)-cholesterol and hypertriglyceridemia were the most prevalent, affecting 88.6% and 34% of the subjects, respectively [25].

Several studies have focused on the role of macronutrients (such as carbohydrates, fat and proteins) and their relation to metabolic syndrome [26,27]. Furthermore, micronutrient (such as vitamins, minerals and trace elements) deficiencies are also related to several debilitating diseases in addition to increased risk of becoming overweight or obese [28]. Abnormal intakes of vitamin C and E and zinc and minerals, like sodium, potassium, calcium and magnesium, favor the occurrence of metabolic syndrome [29,30]. The prevalence of micronutrient deficiencies (especially iron, iodine, zinc and vitamin A and D) are unusually high in the Arab region [31]. Riboflavin deficiency was reported to be high by El-Hazmi and Warsy in different regions of Saudi Arabia [32], while iron deficiency was observed to be common among adults and elderly subjects in Riyadh [33]. Moreover, a study performed by Al-Assaf [34] in healthy adults showed inadequate amounts of vitamin C, B1, B3, B12, B6 and folate in Riyadh, Saudi Arabia.

In lieu of the important role of dietary factors in the prevalence of metabolic syndrome, the present study was designed to investigate the dietary intake patterns of selected dietary nutrients with an emphasis on micronutrients among adult males and females of Riyadh (Saudi Arabia).

## 2. Experimental Section

### 2.1. Study Population

A total of 185 consenting Saudi adults (19–60 years), including males (87) and females (98) (mean age of 35.6 ± 13.2 and 37.6 ± 11.7 years, respectively) were included in this cross-sectional study. The participants were part of an ongoing collaboration between the Biomarkers Research Program (BRP) of King Saud University and the Ministry of Health in Riyadh, Kingdom of Saudi Arabia (RIYADH Cohort). Patient information was obtained from the existing database of more than 17,000 individuals. Patients were recruited randomly from their homes using the cluster sampling strategy. They visited their nearest primary healthcare center (PHCC), which spans the entire Riyadh region. All participants provided written and informed consent prior to inclusion. The population of each PHCC was taken as a cluster, and the allocations of the required numbers of patients were proportional to the populations served by the PHCCs. No expatriates were included in the conduct of this study. Ethical approval was obtained from the Ethics Committee of the College of Science Research Center of King Saud University, Riyadh, Saudi Arabia [35].

#### 2.1.1. Nutritional Evaluation

Dietary intake was assessed using two 24-h dietary recalls conducted by an interviewer using a pre-tested questionnaire. These two recalls were taken at two non-consecutive days. The interviewers were trained dietitians who conducted a face-to face standard interview with the subjects instead of the conventional self-administered questionnaire to ensure validity, reliability and reproducibility. The subjects were asked to report their food consumption, including fluid and diet supplements during the previous day. The questionnaire also included food preparation methods, recipe ingredients and brand names of commercial products. For the verification and estimation of the size of individual portions, the participants were instructed and provided with various food based cues, like graduated food models, a bowl, a cup, a spoon and a measuring cylinder. The physical activity was categorized according to frequency (daily, 3–4, 1–2 times/week, a few times, once/month) and the type of activity (hard, high, middle (1, 2) and low) depending on time duration (˂10, 10–20, 20–30, ˃30 min). The time consumed for each recall was about 15–20 min, and these dietary recalls were the information source of the selected nutrient intakes of the study participants. The quality control of all recall questionnaires was handled and reviewed by an experienced observer to avoid inconsistency and to maintain a uniform decision regarding data entry and analysis. Dietary intakes from 24-h recalls were entered and analyzed for nutrient content using a computerized food database, the US Department of Agriculture Health Tech Software Search and Food Composition for the Middle East [36]. Regarding traditional local foods, the food composition data is complemented by another local study [37]. For selected nutrients, the dietary adequacy was assessed by comparing with the available reference cut-off point values [38,39,40,41]. The selected nutrients for this study included protein, carbohydrates, lipids, calcium, magnesium, zinc and vitamin A, C, D, E and K.

#### 2.1.2. Anthropometric Data and Biochemical Analyses

Subjects were requested to visit their respective primary health care centers (PHCCs) in an overnight fasted state (>10 h) for anthropometry and blood withdrawal by the PHCC nurse and physician on duty. Anthropometry included height (rounded off to the nearest 0.5 cm), weight (rounded off to the nearest 0.1 kg), waist and hip circumference (centimeters) and mean systolic and diastolic arterial blood pressure (BP; millimeters of Hg) (the average of two readings). The body mass index (BMI) was calculated as weight in kilograms divided by height in squared meters. A fasting blood sample was collected and transferred immediately to a non-heparinized tube for centrifugation. Collected serum was then transferred into a pre-labeled plain tube, stored on ice and delivered to the Biomarkers Research Program (BRP) laboratory in King Saud University, Riyadh, Saudi Arabia, on the same day of collection for immediate storage in a −20 °C freezer, pending further analysis. The blood samples were analyzed for fasting glucose and lipid profile including HDL-cholesterol, low-density lipoprotein (LDL)-cholesterol and triglycerides, using a chemical analyzer (Konelab, Espoo, Finland).

#### 2.1.3. Statistical Analyses

Statistical analyses were performed using SPSS version 16.0 (SPSS, Chicago, IL, USA). Groups were compared using analysis of co-variance adjusting for potential confounders, such as age and BMI. Nutrient intakes were expressed as crude, energy adjusted and percentages of total energy using the residual method. Energy-adjusted nutrient intakes were calculated as the residuals from the regression model, with absolute nutrient intake as the dependent variable and total energy intake as the independent variable. The ORs (odd ratios) and their 95% confidence intervals (CIs) for metabolic syndrome were calculated after adjusting for energy, age, physical activity and BMI using logistic regression. The trend for each selected nutrient was established using the highest quartiles as the reference value along with median intake. Based on our analysis, the quartiles covered the extreme cases of deficient dietary intake, enabling us to predict the prevalence of metabolic syndrome. *p*-Values less than 0.05 were accepted to indicate statistically significant differences.

#### 2.1.4. Metabolic Syndrome (Definition)

The prevalence of metabolic syndrome was determined according to the IDF (International Diabetes Foundation) criteria: central obesity as defined by ethnic and sex-specific waist circumference cutoff-points (males, 90 cm; females, 80 cm), plus two of the four other factors: raised triglycerides ≥ 150 mg/dL or 1.7 mmol/L), elevated BP (systolic BP ≥ 130 mmHg or diastolic BP ≥ 85 mmHg), raised fasting plasma glucose (≥5.6 mmol/L or ≥100 mg/dL) and low HDL cholesterol (˂1.03 mmol/L or 40 mg/dL for males; or ˂1.29 mmol/L or 50 mg/dL for females) [42].

## 3. Results

The demographic and anthropometric measurements of the 185 individuals are shown in Table 1. Based on the IDF criteria, 39% of participants had metabolic syndrome, with a higher prevalence in females (55%) than males (24%). Chi-square analysis between genders indicated a significant difference in the MetS distribution (*p* < 0.001) (not mentioned in the table). The dietary intake of selected nutrients in both groups (with and without metabolic syndrome) as compared to reference values is presented in Table 2. The energy-adjusted dietary intake of selected nutrients was lower (except for carbohydrates) in all participants in relation to the respective reference values in both groups (with and without metabolic syndrome). The metabolic syndrome group (male and female) had significantly higher waist circumference (*p* < 0.001), plasma glucose (<0.001) and significantly lower HDL-cholesterol (*p* < 0.001) with no significant difference in physical activity than those without metabolic syndrome (Table 3). With regards to the macronutrients, the females with metabolic syndrome had significantly lower carbohydrate (0.01, 0.007) and protein (0.002, <0.001), expressed as crude intake and total energy adjusted, than those without metabolic syndrome. For selected micronutrients, expressed as crude intake and total energy adjusted, the females with metabolic syndrome had significantly lower values for vitamin A (*p* < 0.001), C (*p* < 0.001), E (*p* < 0.001, 0.009), K (<0.001, 0.01), calcium (<0.001, 0.006), magnesium (*p* ˂ 0.05) and zinc (*p* < 0.001).

The ORs of metabolic syndrome across quartiles of selected dietary nutrients are presented in Table 4 after adjustment for age, BMI, physical activity and total energy intake. The results show that the lowest quartile of total carbohydrate and protein was associated with a nearly three-fold increase in risk, while selected micronutrients indicate that low intakes of vitamin A, C, E, K, calcium, zinc and magnesium carry an increased risk of having metabolic syndrome.

**Table 1:** General characteristics of study participants [Mean ± standard deviation (SD)]. BMI, body mass index; BP, blood pressure; SAD, sagittal abdominal diameter; HDL, low high-density lipoprotein; LDL, low-density lipoprotein.

Parameters	With Metabolic Syndrome		Without Metabolic Syndrome	
	Mean	SD	Mean	SD
*N*	72		113	
Age (years)	38.2	13.4	37.3	12.5
	*Physical activity (%)*			
Daily	28 (39.0)		74 (65.4) *	
3–4 times a week	15 (20.8)		16 (14.1)	
1–2 times a week	16 (22.2)		11 (9.7)	
Few times a month	8 (11.1)		9 (8.2)	
Once in a month	5 (6.9)		3 (2.6)	
BMI (kg/m^2^)	29.8	6.1	27.5	4.0 *
Waist (cm)	90.2	15.2	79.2	14.3 *
SAD (cm)	26.1	17.2	23.1	11.6
Systolic BP (mmHg)	125.4	14.8	121.4	14.5
Diastolic (mmHg)	79.2	8.4	76.5 *	8.4
Glucose (mmol/L)	7.3	0.73	5.7 *	0.64
HDL-cholesterol (mmol/L)	0.83	0.31	0.92	0.24 *
Triglycerides (mmol/L)	1.8	0.25	1.7	0.25 *
LDL-cholesterol (mmol/L)	3.4	0.35	3.2	0.26 *
Total cholesterol (mmol/L)	6.8	1.2	5.1	0.93 *

* *p* < 0.05 *vs.* with metabolic syndrome.

**Table 2:** Daily dietary intakes of selected nutrients of study participants based on reference (ref.) values.

Dietary Intake	AMDR, EAR or AI (UL), M/F	With Metabolic Syndrome				Without Metabolic Syndrome			
		Intake		<Ref. (%)	>Ref. (%)	Intake		<Ref. (%)	>Ref. (%)
		Mean	SE			Mean	SE		
Energy (Kcal)	-	1805.4	90.1	-	-	2100.5	61.7 *	-	-
Carbohydrate (% energy)	45–65 ^†^	67.1	8.0	2.8	76.4	69.3	9.2	0.9	78.8
Protein (% energy)	10–35 ^†^	18.4	5.6	2.8	5.6	20.1	6.2	0.00	3.5
Fat (% energy)	20–35 ^†^	12.5	4.2	77.8	0.00	13.9	5.1	95.6	0.00
Carbohydrate (g)	-	295.9	15.7	-	-	351.6	8.2 *	-	-
Protein (g)	-	85.4	7.3	-	-	96.6	5.8	-	-
Fat (g)	-	26.1	2.1	-	-	28.1	2.2	-	-
Vitamin A (µg RAE)	625/500 ^‡^ (3000) ||	390.4	37.5	37.5	26.4	419.3	41.5 *	34.5	15.9
Vitamin C (mg)	75/60 ^‡^ (2000) ||	50.2	4.1	42.5	8.8	61.2	5.7 *	29.2	20.8
Vitamin D (IU)	650 ^§^	316.0	38.0	91.2	0.00	346.3	28.7	82.4	0.00
Vitamin E (mg)	12 ^‡^ (1000) ||	2.0	0.10	98.7	0.00	2.3	0.08 *	97.2	0.00
Vitamin K (μg PhQ)	90–120 ^§^	12.6	0.95	100	0.00	15.9	0.75 *	100	0.00
Calcium (mg)	800/1000 ^‡^ (2500) ||	569.9	25.5	84.7	9.7	666.7	20.3 *	71.7	15.0
Magnesium (mg)	350/265 ^‡^ (350) ||	287.4	18.3	23.0	54.9	326.8	21.3 *	36.1	36.1
Zinc (mg)	9.4/6.8 ^‡^ (40) ||	6.1	0.38	48.6	22.2	7.1	0.30 *	22.1	46.9

Standard Error (SE); RAE, retinol activity equivalents; IU, international unit; 1 μg = 40 IU; PhQ, phylloquinone; * *p* < 0.05 *vs.* with metabolic syndrome; ^†^ AMDR, acceptable macronutrient distribution range; ^‡^ EAR, estimated average requirement; ^§^ AI, adequate intake (in case EAR not available); **|**| UL, upper limit; adjusted for total energy intake by the residual method of linear regression. The reference values (19–60 years) are equal in both genders, unless otherwise stated as M/F: male/female.

**Table 3:** Daily intake of energy and selected nutrients of the study participants according to gender.

Parameters	Females				Males			
	With Metabolic Syndrome		Without Metabolic Syndrome		With Metabolic Syndrome		Without Metabolic Syndrome	
	Mean SE		Mean SE		Mean SE		Mean SE	
*N*	54		44		21		66	
*Physical activity (%)*								
Daily	22 (41.0)	-	29 (65.9)	-	10 (47.6)	-	42 (63.6)	-
3-4 times a week	12 (22.2)	-	6 (13.6)	-	5 (23.8)	-	10 (15.1)	-
1-2 times a week	10 (18.5)	-	4 (9.1)	-	5 (23.8)	-	7 (10.6) *	-
Few times a month	6 (11.0)	-	4 (9.1)	-	1 (4.8)	-	5 (7.6)	-
Once in a month	4 (7.3)	-	1 (2.3)	-	0	-	2 (3.1)	-
Age (years)	40.1	13.1	35.2	10.3 *	38.7	13.5	32.5	12.9
BMI (kg/m^2^)	31.1	6.8	28.3	4.2 *	29.0	5.4	25.9	3.0 *
Waist (cm)	80.6	7.9	93.9	15.9 *	73.4	8.4	102.0	18.4 *
SAD (cm)	24.7	10.4	20.6	5.7 *	30.0	14.3	24.6	8.7
Systolic BP (mmHg)	122.2	14.6	118.6	17.0	124.9	12.8	118.8	14.0
Diastolic BP (mmHg)	76.7	7.4	75.2	5.9	78.9	6.8	77.2	11.2
Glucose (mmol/L)	6.8	0.72	6.1	0.58 *	5.8	0.80	5.1	0.73 *
HDL-cholesterol (mmol/L)	0.82	0.21	0.99	0.31 *	0.71	0.20	0.93	0.22 *
Triglycerides (mmol/L)	1.3	0.28	1.2	0.25	2.2	0.31	1.6	0.23 *
LDL-cholesterol (mmol/L)	3.4	0.33	3.2	0.23 *	3.6	0.35	3.2	0.31 *
Total cholesterol (mmol/L)	5.2	0.31	4.8	0.27 *	6.5	0.36	4.8	0.28 *
*Dietary Intake*								
Energy (Kcal)	1780.3	102.2	1880	154.3 *	2046.0	106.3	2185.9	102.6
*Carbohydrate (Intake)*								
Crude (g)	291.7	16.5	330.6	8.0 *	335.7	13.6	348.6	8.4
Total energy adjusted (g)	290.3	15.7	329.7	17.4 *	331.8	27.8	354.8	13.2
Total energy (%)	65.5	8.5	69.8	9.9	65.6	8.1	63.8	8.6
*Protein (Intake)*								
Crude (g)	84.5	4.8	92.7	7.3 *	105.1	13.6	91.4	4.8
Total energy adjusted (g)	84.3	5.5	92.2	6.1 *	97.0	9.5	106.8	4.7
Total energy (%)	18.9	5.8	19.7	6.7	20.5	5.7	16.7	6.1
*Fat (Intake)*								
Crude (g)	26.5	1.9	29.3	1.3	25.5	2.6	27.4	2.0
Total energy adjusted (g)	25.8	1.6	30.6	1.8	32.9	2.6	33.7	1.3
Total energy (%)	13.3	6.1	14.0	6.8	11.2	5.5	11.2	5.7
*Vitamin A (Intake)*								
Crude (g)	223.8	35.5	379	42.7 *	523.4	26.4	537.6	42.6
Total energy adjusted (g)	207	32.2	372	40.1 *	513.3	37.5	532.5	41.5
*Vitamin C (Intake)*								
Crude (g)	33.9	5.4	52.1	6.1 *	59.4	6.3	63.2	8.2
Total energy adjusted (g)	47.3	4.6	55.1	5.4 *	62.4	6.7	66.3	8.2
*Vitamin D (Intake)*								
Crude (g)	413.5	63.4	391.2	47.6	298.5	42.8	287.6	29.8
Total energy adjusted (g)	426.1	65.9	397.0	48.4	300.3	43.1	290.2	32.0
*Vitamin E (Intake)*								
Crude (g)	1.6	0.11	2.1	0.12 *	2.1	0.19	2.3	0.10
Total energy adjusted (g)	1.8	0.12	2.3	0.13 *	2.2	0.20	2.4	0.10
*Vitamin K (Intake)*								
Crude (g)	13.0	1.1	17.1	1.6 *	13.1	2.0	14.5	1.8
Total energy adjusted (g)	12.8	1.1	17.1	1.2 *	13.3	1.8	14.9	0.93
*Calcium (Intake)*								
Crude (g)	461.5	33.7	688.0	24.0 *	544.2	31.7	674.9	21.0
Total energy adjusted (g)	534.0	30.2	663.3	33.6 *	626.3	49.8	682.3	25.1
*Magnesium (Intake)*								
Crude (g)	319.1	17.3	414.6	17.8 *	340.1	32.9	364.4	17.7
Total energy adjusted (g)	297.2	17.3	323.4	20.3 *	349.7	34.1	387.9	17.3
*Zinc (Intake)*								
Crude (g)	7.1	0.61	9.6	0.53 *	7.7	0.65	9.3	0.54
Total energy adjusted (g)	2.8	0.44	6.2	0.49 *	8.7	0.74	9.4	0.37

Data adjusted for total energy intake by the residual method of linear regression. * *p* < 0.05 *vs.* with metabolic syndrome.

**Table 4:** Odd ratio (OR) (95% CI) of having metabolic syndrome by quartiles of dietary intake.

Median Intake OR	Q4 (Highest)	Q3	Q2	Q1 (Lowest)
*Carbohydrate*				
Median Intake	487.2	388.4	293.8	197.2
OR (95% CI)	1.0	0.72 (0.29, 1.7)	1.0 (0.41, 2.4)	3.4 (1.4, 8.1) *
*Protein*				
Median Intake	218.9	107.5	75.7	53.9
OR (95% CI)	1.0	0.57 (0.20, 1.6)	0.80 (0.29, 2.2)	3.1 (1.2, 8.3) *
*Fat*				
Median intake	47.0	32.1	20.8	12.7
OR (95% CI)	1.0	0.72 (0.29, 1.8)	1.3 (0.57, 3.2)	1.52 (0.62, 3.7)
*Calcium*				
Median intake	1,003.6	669.6	506.7	340.0
OR (95% CI)	1.0	1.3 (0.46, 3.7)	1.3 (0.42, 3.8)	6.1 (1.9, 19.5) *
*Magnesium*				
Median intake	588.2	417.5	299.0	183.0
OR (95% CI)	1.0	0.62 (0.24, 1.6)	3.1 (1.1, 8.6) *	2.7 (1.0, 7.2) *
*Zinc*				
Median intake	14.0	9.4	7.3	5.0
OR (95% CI)	1.0	0.56 (0.19, 1.6)	0.89 (0.30, 2.6)	5.1 (1.8, 14.5) *
*Vitamin A*				
Median intake	1,816.8	432.3	17.3	11.5
OR (95% CI)	1.0	1.1 (0.77, 1.8)	1.4 (0.83, 1.9)	3.1 (1.5, 5.3) *
*Vitamin C*				
Median intake	139.6	1.56	0.81	0.39
OR (95% CI)	1.0	0.97 (0.61, 1.3)	1.8 (0.86, 2.7)	4.1 (1.6, 8.3) *
*Vitamin D*				
Median intake	666.4	390.6	206.8	137.8
OR (95% CI)	1.0	1.9 (0.84, 1.7)	2.0 (1.0, 6.4)	2.2 (1.6, 8.7)
*Vitamin E*				
Median intake	3.5	2.2	1.6	1.2
OR (95% CI)	1.0	0.85 (0.29, 2.4)	1.2 (0.41, 3.6)	5.9 (2.0, 17.6) *
*Vitamin K*				
Median intake	31.1	15.1	6.3	2.7
OR (95% CI)	1.0	1.9 (0.70, 5.4)	3.3 (0.98, 11.4)	4.1 (1.1, 14.3) *

Data are the odds ratio calculated from logistic regression with dietary nutrient intakes adjusted for age, BMI and physical activity by the residual method of linear regression. * *p* < 0.05.

## 4. Discussion

The study demonstrates an inverse relationship between insufficient dietary micronutrient intake of vitamin A, C, E and K, calcium, zinc and magnesium and metabolic syndrome risk in females than those without metabolic syndrome. For macronutrients, such as carbohydrates and proteins, the same trends were observed in the female group.

The prevalence of metabolic syndrome in Saudi Arabia as demonstrated by Al-Nozha and colleagues [19] shows a higher ratio in females than in males, with 42%, and 37.2%, respectively. It is also noteworthy that due to Westernization of the Saudi Arabian diet, the increased intake of high levels of fat, free sugars, sodium and cholesterol have become much more common in the daily dietary pattern [43,44].

### Metabolic Syndrome and Macronutrients

The data on macronutrient intake and its association with the prevalence of metabolic syndrome are controversial, and to reduce its prevalence, both high-carbohydrate low-fat low-protein diets [45] and low-carbohydrate high-fat high-protein diets have been proposed [46]. By contrast, the present study suggests that a dietary intake of high carbohydrate and high protein shifts the risk towards lower prevalence of metabolic syndrome in females compared to males. Since the present study aimed to investigate the association between metabolic syndrome and selected micronutrients, it was beyond the scope of this study to further discuss the discrepancy between macronutrients and the metabolic syndrome relationship.

Although, it is unclear whether or not factors, like race, ethnicity or gender, can modify the mortality risk associated with metabolic syndrome, it has been suggested that the increased risk of the prevalence of metabolic syndrome in females is indeed due to gender-specific factors [47,48].

Lack of physical activity is strongly associated with metabolic syndrome risk in both adolescents and adults [49,50]. Al-Nozha and colleagues [51] reported a lower level of leisure time physical activity among the Saudis (6.1% in men and 1.9% in women). The subjects included in the present study showed a higher level of physical activity in the control group compared to the metabolic syndrome group. However, this difference disappeared when the subjects were separated according to gender and adjusted for total energy intake.

The dietary pattern in the Middle Eastern region, including Saudi Arabia, has been documented (with a lower consumption of fruits, vegetables, milk and dairy products and an increased consumption of animal products and refined foods in the usual diet) and is more pronounced in adolescent females than in males [52,53]. Dietary supplementation of certain vitamins (C, E, A, CoQ10) and other trace elements is important in balancing antioxidant levels and boosting free-radical scavenging systems that could prevent the evolution toward metabolic syndrome [54]. According to a study performed in USA adults aged over 20 years, there was significantly lower dietary intake of vitamin A with no significant differences for vitamin C and E among participants with and without the metabolic syndrome. Moreover, participants belonging to the metabolic syndrome group had a lower consumption of fruits and vegetables [55]. However, another study in older (≥65 years) Ecuadorians, with micronutrient deficiencies, including vitamin C and zinc, showed a higher prevalence of metabolic syndrome in women (81%) than in men (19%) and demonstrated an inverse relation between vitamin C and E concentrations and metabolic syndrome [56]. The present study demonstrates significantly lower intake of dietary vitamin A, C and E in the female metabolic syndrome group. The decreased intake of these vitamins could be linked to an unhealthier dietary pattern and lower consumption of antioxidants sources, like fruit and vegetables, in Saudi females.

In cross-sectional analysis data from US adults, a higher intake of vitamin K showed a positive effect on the prevalence of metabolic syndrome [57]. The present findings support the above conclusion, since they demonstrate a lower level of dietary vitamin K in females and a higher, but still low, level in males with metabolic syndrome.

The intake of calcium is found to be lower than the recommended dietary reference level in both genders in several regions of Saudi Arabia [34,58]. One study performed by Liu *et al.* [59] demonstrated a lower prevalence of metabolic syndrome with high calcium intake in middle aged and older women. Several other studies also described a favorable inverse association between dietary calcium intake and the risk of having metabolic syndrome in women [60,61]. The present study is in line with the result of Liu *et al.* [59], showing an inverse association of dietary calcium and the prevalence of metabolic syndrome in females. As discussed earlier, the decrease in calcium mainly in females may be attributed to their lower intake of milk and dairy products, which are the main sources of these nutrients.

The dietary magnesium intake is inversely related with the prevalence of metabolic syndrome in men, as well as in women of all age groups [62,63]. The present study supports the above findings and shows that the lowest quartile of dietary magnesium intake is related to a high prevalence of metabolic syndrome. However, this was only in females with metabolic syndrome. The probable cause appears to be a relative deficiency in the main source of magnesium (e.g., green leafy vegetables and dairy products) in Saudi women.

A lower zinc intake has been demonstrated in Saudi Arabia in adolescent females compared to males [58]. An inverse association of high dietary zinc intake and the prevalence of diabetes and metabolic syndrome have been shown in an Indian population [64]. The present study corroborates these findings, showing a positive association between lower dietary zinc intake and the prevalence of metabolic syndrome in the female group.

The authors acknowledge several limitations. The present findings cannot be generalized, due to the small sample size, which is not representative of the overall adult population in Saudi Arabia. Due to the cross-sectional design of this study, the micronutrient levels could not be related to the causality of metabolic syndrome. The limitation of the 24-h dietary recall method for not covering the complete diet of the population seems to be responsible for the low observed total energy intake, which, in turn, is due to the underreporting of total energy intake. However, the present results probably are not affected majorly by this factor, as the selected dietary nutrients were adjusted for total dietary energy intake.

## 5. Conclusions

In conclusion, our study indicates that significantly lower levels of carbohydrates and proteins and a low level of micronutrients, including vitamin A, C, E and K, calcium, zinc and magnesium can help to explain the higher prevalence of metabolic syndrome, especially in adult females. Further research is needed using a larger cohort to confirm the present findings.
